# Chronic Effects of a Wild Green Oat Extract Supplementation on Cognitive Performance in Older Adults: A Randomised, Double-Blind, Placebo-Controlled, Crossover Trial

**DOI:** 10.3390/nu4050331

**Published:** 2012-05-03

**Authors:** Rachel H. X. Wong, Peter R. C. Howe, Janet Bryan, Alison M. Coates, Jonathan D. Buckley, Narelle M. Berry

**Affiliations:** 1 Nutritional Physiology Research Centre, Sansom Institute for Health Research, University of South Australia, GPO Box 2471, Adelaide, South Australia 5001, Australia; Email: rachel.wong@mymail.unisa.edu.au (R.H.X.W.); janet.bryan@unisa.edu.au (J.B.); alison.coates@unisa.edu.au (A.M.C.); jon.buckley@unisa.edu.au (J.D.B.); narelle.berry@unisa.edu.au (N.M.B.); 2 School of Psychology, Social Work and Social Policy, University of South Australia, GPO Box 2471, Adelaide, South Australia 5001, Australia

**Keywords:** *Avena sativa*, wild green oat extract, Neuravena, cognition, attention-concentration, older adults

## Abstract

Background and aim: Preliminary evaluation of a wild green oat extract (WGOE) (Neuravena^®^ ELFA^®^955, Frutarom, Switzerland) revealed an acute cognitive benefit of supplementation. This study investigated whether regular daily WGOE supplementation would result in sustained cognitive improvements. Method: A 12-week randomised, double-blind, placebo-controlled cross-over trial of WGOE supplementation (1500 mg/day) *versus* placebo was undertaken in 37 healthy adults aged 67 ± 0.8 years (mean ± SEM). Cognitive assessments included the Stroop colour-word test, letter cancellation, the rule-shift task, a computerised multi-tasking test battery and the trail-making task. All assessments were conducted in Week 12 and repeated in Week 24 whilst subjects were fasted and at least 18 h after taking the last dose of supplement. Result: Chronic WGOE supplementation did not affect any measures of cognition. Conclusion: It appears that the cognitive benefit of acute WGOE supplementation does not persist with chronic treatment in older adults with normal cognition. It remains to be seen whether sustained effects of WGOE supplementation may be more evident in those with mild cognitive impairment.

## 1. Introduction

*Avena sativa* L. (oats), in its various forms and extracts, has been traditionally used for its physical and psychological fortifying properties for centuries. The purported benefits include reduced risk of heart disease, mild anti-depressant effects, increased ability to cope with stress, reduced anxiety [1,2], anti-inflammatory properties [3] and relief of skin irritation [4]. Although some of these benefits await rigorous scientific substantiation, recent studies suggest that supplementation with an oat extract from green oats, Neuravena^®^ (EFLA^®^955, Frutarom, Switzerland), can acutely improve mental function in humans [5,6]. A single oral dose of 2500 mg wild green oat extract (WGOE) in healthy humans significantly increase in theta electric brain activity in the left frontotemporal region during a concentration task, measured by computer aided topographical electroencephalometry, compared with a 1250 mg dose or a placebo. The significant increase in theta brain activity, an indicator of cognitive alertness, two hours post supplementation with WGOE [6], suggests the potential for a cognitive benefit. This potential cognitive benefit was recently supported in an acute intervention in our laboratory, where consumption of a single dose of WGOE (1600 mg) resulted in a reduction in the number of errors made in the Stroop colour-word test in older adults with below average cognition (DEMTECT score 9–16) [5], indicative of an improvement in attention and the ability to ignore distraction. 

The mechanism of effect of WGOE is currently unknown. *In vitro* bioassays of WGOE indicated inhibitory effects on monoamine oxidase B (MAO-B) and phosphodiesterase-4 (PDE4). Effects of the latter may have a vasodilator benefit in the central nervous system, promoting potential enhanced vasodilatation in the cerebral vessels, thus aiding cognitive function [7]. In addition, avenanthramides (bioactive compounds unique in oats) have been shown to enhance nitric oxide (NO) production in human aortic smooth muscle cells [8] and suppress inflammatory cytokines by inhibiting nuclear factor κB (NF-κB) activation [3]; both mechanisms may elicit vasodilation in the cerebral arteries. Mozolic *et al*. [9] recently showed that an enhanced ability to concentrate during a visual and auditory attention task was linked to an improvement in resting cerebral blood flow in healthy older adults. Indeed, cerebral blood flow is reduced in those with mild cognitive impairment [10]. However the association between enhanced cerebral perfusion and improved cognitive performance has yet to be established, especially in healthy older adults with normal cognition.

Whilst interventions with some nutrients have demonstrated acute cognitive improvements with a single dose of supplement [11,12], these nutrients may not necessarily have persistent cognitive benefits with daily consumption over longer periods (*i.e.*, 12 weeks) [13,14]. In terms of acute cognitive effects, it is not clear how long these effects persist for as the time between consuming the last supplement dose and the administration of cognitive tests in the literature is unclear. To assess a nutrient’s potential for improving human cognition over the longer-term; it is preferable for its effects to result from persistent, rather than transient, physiological changes. Therefore in this study, we aimed to expand on our previous work with acute WGOE supplementation [5] to determine if daily consumption of a WGOE would result in sustained cognitive improvements in healthy older adults. 

## 2. Experimental Section

### 2.1. Study Design and Participants

A 12-week randomised, double-blind, placebo-controlled cross-over human dietary intervention trial with 1500 mg per day of WGOE or placebo was conducted at the Nutritional Physiology Research Centre, University of South Australia, Adelaide, in accordance with principles of Good Clinical Practice. The study was approved by the University of South Australia Human Research Ethics Committee and registered with the Australia and New Zealand Clinical Trials Registry (ACTRN12610000012077). Healthy males and females aged over 60 years were recruited from the public and provided written, informed consent prior to screening. 

During an initial screening visit, participants underwent a series of physiological and cognitive assessments to determine their eligibility before formally enrolling into the trial. Participants were assigned a study ID in the order of their enrolment. Participants were excluded if they met any of the following conditions: were unable to read and write English; unable to distinguish the colours “red”, “green”, “blue” and “yellow” without difficulty; were taking cognitive enhancers, anti-cholinergic medication or mood altering medications; had a history of serious head injury, diagnosed and/or treated mental illness, alcoholism, stroke or neurological condition; had cardiovascular disease or renal disease or diabetes or gastrointestinal disease or dyslexia; smoked or used nicotine replacement therapy; had changed antihypertensive medication in the preceding three months or were likely to do so during the trial; consumed more than one serve of oats (including oat products) on average per day. 

Additional exclusion criteria determined at screening included suspected dementia [15] (DEMTECT score < 9), or resting supine blood pressure (BP) > 160/100 mmHg. During the intervention, participants were instructed to maintain their usual dietary habits and level of physical activity. Outcome assessments obtained during screening were for familiarization purposes; therefore only participant demographics are reported. 

### 2.2. Sample Size

This study was powered based on the effect size (*d* = 0.62) for the Stroop colour-word test uncorrected error responses from our acute study with WGOE [5] in which a difference of 2.2 ± 3.5 (mean ± SD) errors was observed between 1600 mg WGOE and placebo. Assuming a similar effect size for the present study, 23 participants (male and/or female) were needed to give 80% power for statistical significance at alpha level of 0.05. Due to the length of intervention, 42 participants were recruited to allow for dropouts. 

### 2.3. Supplements

The WGOE (Neuravena^®^ EFLA^®^955) is an extract of a variety of* Avena sativa* L., wild green oat herb. Extraction method has been previously described [6]. Potentially bioactive constituents of oat herb include avenanthramides, saponins, phytoalexin and flavonoids such as vitexin and isovitexin (Frutarom Switzerland Ltd Bibliographic Dossier). 

The WGOE and placebo supplements used in this intervention were supplied by ASK Intercity Co. Ltd., Japan, as authorised by Frutarom Switzerland Ltd., in powder form and pressed into 315 mg capsules. Each capsule of WGOE contained 300 mg of active ingredient and 15 mg of calcium stearate. Placebo capsules contained microcrystalline cellulose (154.5 mg), lactose (154.5 mg), cacao brown (4 mg) and safflower colour (2 mg). These ingredients are not known to have any physiological or psychological effects. WGOE and placebo capsules were near identical in appearance and presented in opaque bottles. 

### 2.4. Intervention Protocol

**Figure 1:**
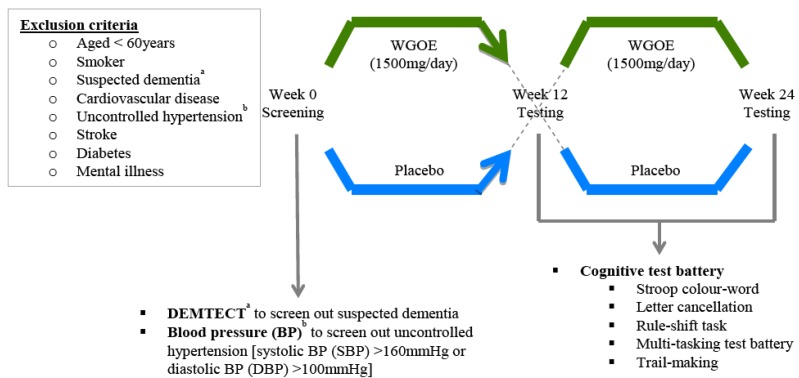
Outline of study design. During the screening visit (Week 0), participants underwent physiological and cognitive assessments including supine blood pressure (BP), DEMTECT and Stroop colour-word test to ensure their suitability for the study before they were randomized to a treatment arm. A complete battery of cognitive assessments was conducted in Week 12 before participants switched over to the alternate treatment and repeated the same assessment battery in Week 24.

Cognitive assessments were conducted in Week 12 and repeated in the same order in Week 24 of the trial (Figure 1). To reduce the possible influence of foods and medications on cognitive outcomes, participants arrived at the research centre at least 4 h fasted (no food, medication, fluids except water) and at least 18 h after taking their last treatment doses. One investigator was responsible for performing all cognitive assessments in the order listed below.

#### 2.4.1. Stroop Colour-Word Test

The Stroop colour-word test is a cognitive test that requires participants to firstly read the names of colours printed in incongruent coloured ink (trial 1), then name the colours in which they are printed (trial 2) as quickly as possible without making errors. The test measures the individual’s ability to suppress task-irrelevant responses (*i.e.*, the tendency to read the colour name rather than name the colour) and ability to maintain attention and concentration [16]. The Stroop interference score was calculated as the time taken to name colours (trial 2) divided by the time taken to read colour names (trial 1). A higher Stroop interference score reflects the degree of interference afforded by suppressing the habit of reading words in order to name colours; thus higher scores reflect poorer performance. Uncorrected errors during trial 2 would reflect failure of inhibition [16].

#### 2.4.2. Letter Cancellation Task

The Letter Cancellation test comprises a 32 × 45 matrix of random capital letters. Participants are required to search for the letters “F” and “L” and cross them out as quickly as possible. Higher performance (less time taken and fewer errors) reflected higher attention and ability to ignore distraction. Strong test-retest reliability has been reported (*r* = 0.93) and moderate to strong convergent validity with other cognitive assessments that measure attention and concentration [17].

#### 2.4.3. Rule-Shift Task

The rule shift task required the participants to change their responses when the rules of the task changed after habituating to the previous rule. The task utilises four sets of 26 images of playing cards presented on a computer screen and participants were asked to respond to the colour of the suit of the cards. For example, in the first trial, participants were instructed to respond “yes” if the suit of the card was red and “no” if black, followed by a second trial in which they responded “no” if red and “yes” if black. A higher score, reflected by shorter time taken to complete the task and fewer errors, reflects higher mental switching ability and to inhibit task-irrelevant responses [18]. 

#### 2.4.4. Computerised Multi-Tasking Test Battery

In the multi-tasking computerized battery (Purple Research Solutions, UK) [19], participants were presented with four tasks simultaneously on a computer screen to which they were required to attend and respond quickly to each task equally for 5 min. In one task window, participants scan through a 4 × 4 matrix of numbers and select the highest digit. Upon successful completion, a fresh matrix of numbers appears. Another task comprises of a red dot drifting outwards from the centre of a circle. Before it falls outside, the participant must press the “reset” button located at the bottom of task window. In another task window, six red bars will rise at different rates. As soon as one of the red bars reaches the top, a “warning sign” flashes and the participant must select the bars in numerical sequence. Once completed, the task restarts itself. The fourth task window consists of four colour blocks (red, blue, green and yellow) and a name of colour printed in incongruent coloured ink. The participants are required to click on the colour block that corresponds with the colour of the text, not the word. Points are awarded for successful completion of each task. Participants are negatively scored if mistakes are made and if they fail to attend of a task within a randomly assigned allocated time. These tasks therefore assessed divided attention, concentration and the ability to ignore task-irrelevant information. This cognitive assessment has been used previously in studies that measure the effects of bioactive nutrients on cognitive performance and has the sensitivity to detect treatment differences [20,21]. A higher score reflects their ability to multi-task with speed and accuracy. 

The multi-tasking test is a computer-based cognitive assessment tool, which may have a practice effect and/or some older adults were unaccustomed to using a computer mouse. Therefore, participants were familiarized with the computerized multi-tasking test battery during the screening visit, thereby reducing the practice effect during the intervention. 

#### 2.4.5. Trail-Making Task

The Trail-Making test is comprised of two parts that require participants to first draw lines to join numbers in ascending order. In the second part, participants are instructed to alternate (in ascending sequence) with letters and to search for the numbers and letters from a random array (*i.e.*, 1-A-2-B-3-C-4-D, *etc*.). As such the task assesses mental switching, the ability to ignore distraction and to inhibit task irrelevant information [22].

### 2.5. Statistical Analysis

Statistical analyses were performed using IBM SPSS Version 18.0 (New York, United States). The primary outcome was the number of uncorrected errors made during the colour-naming task on the Stroop colour-word test on which the study was powered. The effect of treatment was determined by within-individual comparison of the values obtained at the end of each treatment period using a paired *t*-test. Secondary outcomes were other improvements in cognitive performance as measured by the Stroop colour-word test, letter cancellation, rule-shift, trail-making and computerized multi-tasking test battery. Each cognitive outcome measurement (Stroop colour-word test, Letter cancellation, Rule-shift, computerized multi-tasking test battery and Trail-Making) was examined individually and then also collectively in the form of a composite attention-concentration index, which was obtained by summing the Z-scores of all the cognitive assessment data. A sequential Bonferroni adjustment was made to allow for multiple comparisons. Allowing for the fact that several assessments of the cognitive test battery were interdependent, the critical P-value for the most significant outcome was reduced from <0.05 to <0.005 (*i.e.*, <0.05/10). All data are presented as mean ± SEM, unless stated otherwise. 

## 3. Results

### 3.1. Participant Characteristics

Of the 42 participants who were enrolled, 37 completed the two assessment time points in the 24-week intervention and were included in the data analysis. Three of the five who withdrew were taking WGOE supplement—one was hospitalized and treated for angina, one had a change of BP medication and one withdrew due to gastrointestinal discomfort. Two participants (one had newly diagnosed breast cancer and one had gastrointestinal discomfort) were taking the placebo at the time of withdrawal. 

**Table 1:** Participant characteristics at time of screening (data expressed as mean ± SEM). Blood pressure values were determined at screening.

Participant characteristics at enrolment	
Number of Participants (Males/Females)	37 (25/12)
Age (years)	67 ± 0.8
Height (m)	1.71 ± 0.02
Mass (kg)	77.4 ± 2.1
Body Mass Index (kg/m^2^)	26.4 ± 0.6
DEMTECT Score	17.5 ± 0.2
SBP (mmHg)	127 ± 2
DBP (mmHg)	71 ± 1

Table 1 shows the participant characteristics. Participants in this study were healthy, normotensive but slightly overweight. They were also of normal cognition with DEMTECT scores ranging between 14 and 19 out of a maximum score of 19, with a median score of 18 (mild cognitive impairment 9 to 12 points, suspected dementia <9 points) [15]. During the intervention, the concomitant medications and dietary supplement use by participants included cholesterol-lowering (19%), anti-hypertensives (16%), gout (5%), fish oil (35%), calcium (14%) and multi-vitamins (17%). Compliance with the intervention was greater than 99% for both intervention arms (99 ± 0.8% for treatment dose; 100 ± 0.6% for placebo).

### 3.2. Cognitive Outcomes

Table 2 shows the results of each component of the cognitive assessments. No significant differences were observed in any of the individual tests or in the overall attention-concentration index. 

**Table 2:** Cognitive assessments. Data are presented as mean ± SEM, *N* = 37. A higher score reflects poorer performance with the exception of multitasking where it reflects better performance.

	WGOE	Placebo	ΔWGOE − Placebo	*t*	*P*
**Stroop Colour Word**					
Word time (s)	92.4 ± 2.9	94.1 ± 2.9	−1.8 ± 1.1	−1.53	0.13
Colour time (s)	203.1 ± 8.9	198.7 ± 7.7	4.5 ± 2.8	1.82	0.08
Word uncorrected errors	0.5 ± 0.3	0.7 ± 0.5	−0.1 ± 0.6	−0.23	0.82
Colour uncorrected errors	2.2 ± 0.5	2.1 ± 0.4	0.1 ± 0.4	0.37	0.72
Word corrected errors	0.2 ± 0.1	0.3 ± 0.1	−0.1 ± 0.1	−0.70	0.49
Colour corrected errors	2.4 ± 0.4	2.3 ± 0.4	0.1 ± 0.4	0.14	0.89
Interference score	2.2 ± 0.1	2.1 ± 0.1	0.1 ± 0.04	2.26	0.04
**Letter Cancellation**					
Time (s)	392.4 ± 11.5	395.9 ± 12.1	−3.5 ± 9	−0.39	0.70
Errors	13.1 ± 1.5	14.7 ± 1.8	−1.6 ± 1.3	−1.21	0.23
**Rule Shift**					
Rule shift 1 time (s)	25.2 ± 0.7	25.6 ± 0.9	−0.4 ± 0.8	−0.49	0.63
Rule shift 1 errors	0.1 ± 0.05	0.1 ± 0.05	0.0 ± 0.1	0.00	1.00
Rule shift 2 time (s)	27.0 ± 1.0	26.6 ± 0.9	0.4 ± 1.0	0.42	0.68
Rule shift 2 errors	1.2 ± 0.5	1.1 ± 0.4	0.1 ± 0.6	0.23	0.82
Rule shift interference 1	1.1 ± 0.03	1.1 ± 0.03	0.02 ± 0.04	0.51	0.61
Rule shift 3 time (s)	37.5 ± 1.6	36.2 ± 1.2	1.3 ± 1.4	0.92	0.37
Rule shift 3 errors	1.6 ± 0.3	1.3 ± 0.3	0.3 ± 0.4	0.60	0.55
Rule shift 4 time (s)	46.2 ± 2.2	46.5 ± 2.3	−0.3 ± 2.6	−0.13	0.90
Rule shift 4 errors	5.8 ± 1	6.4 ± 1.2	−0.6 ± 1.4	−0.44	0.67
Rule shift interference 2	1.2 ± 0.05	1.3 ± 0.1	−0.05 ± 0.1	−0.61	0.55
**Trail Making**					
Condition 1 time (s)	42.5 ± 2.4	40.1 ± 2.1	2.4 ± 2.3	1.04	0.30
Condition 1 errors	0.2 ± 0.1	0.1 ± 0.1	0.1 ± 0.1	1.14	0.26
Condition 2 time (s)	91.9 ± 5.2	92.5 ± 8.1	−0.6 ± 5.9	−0.11	0.92
Condition 2 errors	0.7 ± 0.1	0.8 ± 0.2	−0.1 ± 0.1	−0.40	0.69
Interference	2.3 ± 0.1	2.4 ± 0.2	−0.1 ± 0.1	0.73	0.47
**Multitasking Score**	616.1 ± 74.7	602.8 ± 64.2	13.2 ± 41.8	0.32	0.75
**Attention-concentration Index**	3.0 × 10^−5^	1.5 × 10^−4^	−1.2 × 10^−4^	0.00	1.00

## 4. Discussion

This is the first study to investigate the effects of chronic WGOE supplementation (1500 mg per day for 12 weeks) on cognitive performance in older adults. Our WGOE dose of 1500 mg/day for 12 weeks was well-tolerated by the study participants. We found no significant change in the primary outcome measure, *i.e.*, the number of errors made in the colour-naming trial (trial 2) of the Stroop colour-word test. There were no significant effects on any other measures of cognitive performance or in the attention and concentration index following chronic WGOE supplementation in this healthy sample of older adults. 

In our recent acute WGOE supplementation study, there was a significant reduction in the number of errors made during the Stroop colour-word test following acute supplementation with 1600 mg of WGOE compared with placebo [5]. Importantly, in our previous study those participants suspected of having mild cognitive impairment (DEMTECT score between 9 and 12, *n* = 7) demonstrated the biggest improvement in cognitive function. The remaining 29 participants had DEMTECT scores between 12 and 16 out of the maximum 19. In contrast, participants in the present study comprised healthy older adults with “normal” cognitive function who may have been less likely to show significant improvements in cognition. Furthermore, the median DEMTECT score in our study participants was 18 out of the maximum 19, which may represent a sampling bias due to the recruitment nature of volunteering for such dietary intervention with an aim to improve cognitive performance through supplementation. As such, our cohort is likely to fall within the upper deciles of cognitive performance in their age-group, thus reducing the likelihood of detecting statistical and/or clinically significant improvements with chronic WGOE supplementation. Future work with WGOE should include a sample with a range of DEMTECT scores. Nonetheless, albeit a small sample size, daily consumption of blueberry juice had beneficial effects on memory in older adults with mild cognitive impairment [23]. Perhaps the cognitive enhancing benefit of a single dose of WGOE seen in the acute study [5] may be due to a direct, transient increase in theta brain wave activity during a concentration task, as previously observed [6]. The sustained effect of WGOE on human brain wave activity has not been explored. 

Whilst there were no cognitive improvements following daily WGOE supplementation, we did not see any decline in cognitive performance after each arm of treatment. The rate of cognitive decline of older adults over time can be estimated by examining cognitive change in control groups. For example, a 12-month exercise intervention found significant within-individual decline in cognition in a control group of healthy older adults (65–74 years old), as measured using the Mini Mental State Examination (MMSE), even though the MMSE scores of the control group remained well-within the range of normal cognition after a year [24]. Considering the decline in cognitive performance with age [22] (and with time [24]), regular daily consumption of WGOE may be beneficial for attenuating age-related cognitive decline in healthy older adults.

The cognitive assessment battery used in this study measured only one construct of cognition—attention and concentration. Whilst we recognised the anticipated age-related decline in memory [25], it would not have been feasible to include all domains of cognition in our study protocol as increasing the number of outcome variables would have increased the risk of Type 1 error within the sample size available. Our rationale for measuring this construct was based on our previous observation [5] of an acute benefit of WGOE on the Stroop colour-word test, a test designed to assess attention and concentration under duress [16]. Thus to increase the confidence of our cognitive tests, we selected a test battery focusing on assessing attention-concentration to increase the confidence of the findings. Cluster analysis examining the patterns of cognitive function across the lifespan revealed a discrepancy in cognitive performances between various cognitive domains among older adults (*i.e.*, intact memory but reduced performance in attention) [22]. Given the heterogeneity in the decline of various cognitive domains among age-matched individuals we cannot rule out the possibility that other cognitive constructs such as memory, language, arithmetic and motor performance may be improved with chronic WGOE supplementation and therefore should be explored in future studies.

In this study, cognitive tests were administered at least 18 h after participants consumed their last supplement dose. Thus, we were assessing whether chronic daily WGOE consumption would elicit a sustained cognitive benefit. Daily supplementation with 1500 mg of WGOE did not enhance cognitive performance in our study sample under our testing conditions. Preclinical evidence of WGOE indicated inhibitory effects on MAO-B, an enzyme that metabolises dopamine (a neurotransmitter in the central nervous system that regulates cognitive function [26]), and PDE4, which may have vasodilatory effects on cerebral arteries to enhance blood flow [7]. The short and long term effects of WGOE on dopamine levels and cerebral perfusion (together and independently) in humans and its relationship with human cognition is currently unknown. At this stage, we are unable to speculate if these are mechanisms by which WGOE may influence cognitive function. Nonetheless, it has been shown that avenanthramides (a polyphenol unique to oats) can be detected in the human circulation for up to 10 h following a serve of skim milk containing 1000 mg of avenanthramides [27]. In rats, avenanthramides accumulate in tissues for up to 12 h following oral ingestion [28]. The amount of avenanthramides in our 1500 mg dose of WGOE and its bioavailability and tissue accumulation following WGOE ingestion in humans are not known. Future research on the optimal quantity of avenanthramides in WGOE and the duration of supplementation for enhancing cognition is warranted. Additionally, the anti-inflammatory properties of avenanthramides in oats [3] may be helpful for those with chronic conditions with underlying inflammation such as obesity or Metabolic Syndrome. Considering the relationship between inflammation and cognitive impairment [29], WGOE should also be evaluated in these populations.

## 5. Conclusion

In summary, 12 weeks of daily WGOE supplementation did not alter cognitive performance in our healthy and cognitively-normal older adults. The chronic effects of WGOE supplementation should be extended to those with, or at risk of, mild cognitive impairment and evaluated in other domains of cognition.
